# Facile synthesis of MoO_2_/CaSO_4_ composites as highly efficient adsorbents for congo red and rhodamine B†

**DOI:** 10.1039/c7ra11292k

**Published:** 2018-01-04

**Authors:** Xin-Jian Jia, Jinshu Wang, Junshu Wu, Weili Teng, Bingxin Zhao, Hongyi Li, Yucheng Du

**Affiliations:** Key Laboratory of Advanced Functional Materials for Ministry of Education, College of Materials Science and Engineering, Beijing University of Technology Beijing 100124 China wangjsh@bjut.edu.cn

## Abstract

A novel rod-shaped MoO_2_/CaSO_4_ composite was prepared by using hexa-ammonium molybdate and flue gas desulfurization gypsum *via* a simple mixed-solvothermal route. In this composite, CaSO_4_ matrices are decorated with MoO_2_ nanoparticles, and non-structural mesopores are formed *via* particle packing. Moreover, it displays an excellent adsorption capability towards anionic congo red (CR) and cationic rhodamine B (RhB). The adsorption quantities per unit mass and removal efficiencies of the two dyes are significantly influenced by adsorbent dose, solution pH, and temperature. The adsorption isotherm data can be best fitted by the Langmuir model, and the calculated maximum adsorption quantities at 303.5 K are 853.54 mg g^−1^ for CR and 86.38 mg g^−1^ for RhB, respectively, which are superior to other common adsorbents. The corresponding kinetic data can be well matched with the pseudo-second-order model. Additionally, the CR adsorption is an exothermic process, while the RhB adsorption is an endothermic process. Both of them are multi-step chemisorption processes influenced by surface adsorption and intra-particle diffusion. This MoO_2_/CaSO_4_ composite can be applied as an alternative adsorbent for removing organic dyestuffs from printing and dyeing wastewater.

## Abstract 2

A new kind of rod-shaped MoO_2_/CaSO_4_ composite, in which MoO_2_ nanoparticles are supported on the surface of CaSO_4_ matrices, was prepared *via* a mixed-solvothermal method for efficient removal towards congo red and rhodamine B.

## Graphical abstract

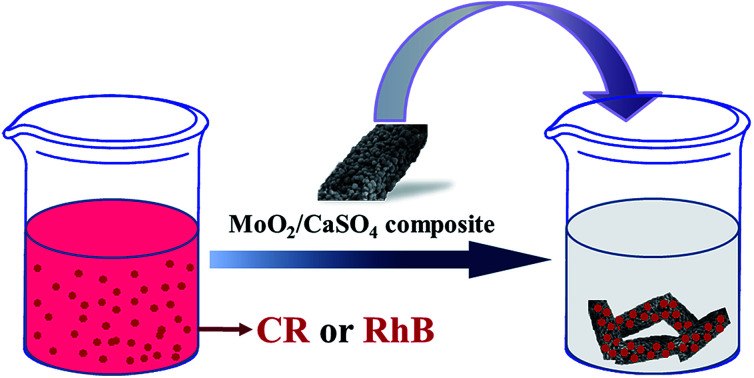

## 1. Introduction

Composite materials, consisting of two or more components, can display more attractive physical and chemical properties than homogenous monolithic materials. They are extensively used in the catalyst, pharmaceutical, building construction, energy and aerospace industries.^1–5^ Although numerous composites with various functions have been reported during the last few decades, the preparation and functionalization of them will continue to be a focus for future research.

In recent years, a series of socio-environmental issues have occurred owing to the discharge of dye-containing effluents without any effective treatment. Many techniques including flocculation, adsorption, membrane separation, ion exchange, photocatalysis, chemical oxidation and biodegradation have been proposed to eliminate dyestuffs from polluted water.^6,7^ However, adsorption is undoubtedly recognized as one of the most popular approaches owing to its simplicity and flexibility, as well as the availability of various adsorbents.^8^ Up to now, a variety of materials have been tested as adsorbents for the purification of dye-containing wastewater.^9,10^ Among these materials, composites are attracting great attention from scientists because of their excellent adsorption capacity. For instance, Luo *et al.* described a magnetic chitosan/graphene oxide composite which could adsorb methylene blue (MB) from a simulated sewage system.^11^ Shen *et al.* synthesized a Fe_3_O_4_/Cu_2_O/PANI composite with well adsorption ability towards congo red (CR) and methyl orange (MO).^12^ Yang *et al.* showed the selective adsorption ability of a polyoxometalate-based metal–organic framework composite towards MB in aqueous solution.^13^ Nevertheless, the widespread use of these absorbents remains restricted due to the complicated preparation process and single function towards the same type of dyes. Therefore, the development and application of novel composite adsorbents, especially those which can effectively remove both cationic and anionic dyes, will still be full of challenges.

As one of the most common industrial by-products, flue gas desulfurization (FGD) gypsum is mainly composed of calcium sulfate dihydrate (CaSO_4_·2H_2_O) and can be widely used as a building material, a soil conditioner, or an additive in cement in place of natural gypsum.^14^ Its purification and transformation into high value-added products have been elaborated in previous work.^15,16^ So far, however, the utilization rate of FGD gypsum is still very low, which means that its comprehensive utilization needs to be further developed. More recently, molybdenum oxides have become one type of the most promising transition metal oxides due to their emerging applications in the fields of optoelectronics, catalysis, medicine and gas sensors.^17–20^ Moreover, molybdenum trioxide (MoO_3_) has been reported as an individual adsorbent or part of a composite adsorbent for sewage treatment.^21–23^ Nevertheless, the synthesis of a composite material loaded with molybdenum dioxide (MoO_2_) on anhydrite (CaSO_4_) matrices and its adsorption behavior have not been reported.

In this work, a kind of rod-shaped MoO_2_/CaSO_4_ composite, in which MoO_2_ nanoparticles are supported on surface of CaSO_4_ matrices, was successfully fabricated with FGD gypsum and hexa-ammonium molybdate ((NH_4_)_6_Mo_7_O_24_·4H_2_O, HM) by a mixed-solvothermal method. The micromorphology and phase structure of the as-prepared composite were investigated, and its adsorption ability towards cationic and anionic dyes in aqueous solution was also determined. The adsorption quantities of the dyestuffs onto this composite material were then evaluated by taking into account the effects of adsorbent dose and solution pH, as well as temperature. All results of the adsorption measurements were linearly fitted according to adsorption isotherm and kinetic models. Furthermore, the mechanism of dye adsorption was further discussed.

## 2. Experimental section

### 2.1. Chemicals

The FGD gypsum was obtained from Zhongwei power plant (Ningxia, China). Analytical grade sodium hydroxide (NaOH), hydrochloric acid (HCl, 37.5%) and anhydrous ethanol (EtOH, ≥98%) were purchased from Beijing Chemical Works (Beijing, China) and used as received without further purification. Analytical grade HM, CR, MO and rhodamine B (RhB) were supplied by Sinopharm Chemical Reagent Co., Ltd. (Shanghai, China). All solutions were prepared from deionized water with a resistivity of 18 MΩ cm unless otherwise specified.

### 2.2. Composite preparation

The MoO_2_/CaSO_4_ composite was prepared *via* a mixed solvothermal route. A typical synthetic procedure involving two steps is described in detail as follows: (1) firstly, 3.0 g of FGD gypsum was added to 40 mL of 5.0 M HCl with continuous stirring. After that, the mixture was transferred into a 100 mL Teflon-lined stainless-steel autoclave, which was then kept at 110 °C for 6 h and cooled down to room temperature naturally. A pale yellow filtrate was obtained by filtration, and then it was crystallized by dropwise adding the same volume of EtOH. Finally, the white precipitate was filtered, washed with deionized water twice, and then dried in an oven at 80 °C for 6 h. (2) Firstly, 0.8 g of HM was dissolved into a 20 mL of HCl/EtOH mixed solution under stirring, in which the volume ratio of 3.0 M HCl to EtOH was 3 : 1, followed by the addition of 0.5 g of the purified FGD gypsum. Subsequently, the mixture was transferred into a 50 mL Teflon-lined stainless-steel autoclave, which was then maintained at 180 °C for 15 h and cooled down to room temperature naturally. Finally, the black product was filtered and dried at 80 °C overnight after being washed with EtOH three times.

### 2.3. Characterization

Scanning electron microscopy (SEM) images were acquired by a Hitachi S-4800 SEM coupled with an energy-dispersive X-ray (EDX) detector. Transmission electron microscope (TEM) images were captured by a FEI Tecnai G^2^ F30 TEM combined with selected area electron diffraction (SAED) at an acceleration voltage of 300 kV. Powder X-ray diffraction (PXRD) patterns were measured on a Bruker D8 Advance X-ray diffractometer with Cu-Kα irradiation (*λ* = 1.54056 Å), and the corresponding data were collected in the 2*θ* range of 5° to 70° with a step size of 0.02°. The accelerating voltage was set at 40 kV with an applied current of 40 mA. Brunauer–Emmett–Teller (BET) specific surface areas were determined using a Micromeritics ASAP2020 instrument according to N_2_ adsorption–desorption isotherms at 77 K. Attenuated total reflectance Fourier transform infrared (ATR-FT-IR) spectra were recorded with a Bruker TENSOR27 spectrometer over the wavenumber range between 4000 and 400 cm^−1^ at a resolution of 4 cm^−1^. X-ray photoelectron spectroscopy (XPS) experiments were conducted on a PHI Quantera SXM X-ray photoelectron spectrometer equipped with a monochromatic Al-Kα X-ray source (*hν* = 1486.6 eV). Survey spectra were taken with a pass energy of 280.0 eV and a step size of 1.000 eV. High-resolution spectra were recorded with a pass energy of 55.0 eV and a step size of 0.100 eV. X-ray fluorescence (XRF) analyses were carried out on a Shimadzu XRF-1800 spectrometer. Ultraviolet-visible (UV-vis) absorption spectra were measured with a Shimadzu UV-2550 spectrophotometer at 498 nm, 554 nm and 464 nm, respectively, so as to confirm the concentrations of CR, RhB and MO in supernatant.

### 2.4. Adsorption measurements

Three dyestuff solutions with high concentrations (5000 mg L^−1^ CR, 1000 mg L^−1^ RhB and 500 mg L^−1^ MO) were prepared as standard stock solutions in advance, and then they were diluted into desired concentrations for subsequent experiments. The adsorptive experiments of this MoO_2_/CaSO_4_ composite towards various synthetic dyes were carried out by adding 40 mg sample into 20 mL of the dye solutions with different initial concentrations. In view of the influences of adsorbent dose and solution pH on dye removal efficiency, the dosage was varied from 0.5 to 5.0 g L^−1^, and the pH adjusted using 0.1 M HCl and 0.1 M NaOH was changed from 2.0 to 12.0. The adsorption equilibrium processes taken place in dye-containing solutions were investigated over the concentration range between 100 and 4000 mg g^−1^. The contact time was kept in the range from 0 to 12 h. At a scheduled time point, the suspension was centrifuged for 5 min at 10 000 rpm, and 10 mL of the supernatant was extracted to determine the residual concentration of a dyestuff by UV-vis spectrometry. All tests were performed in batch mode at 303.5 K and conducted in triplicate unless otherwise specified.

The adsorption capacity (*q*_e_, mg g^−1^) and removal efficiency (*R*_e_) at equilibrium, as well as the adsorption quantity at time *t* (*q*_*t*_, mg g^−1^) of a synthetic dye onto this MoO_2_/CaSO_4_ composite were evaluated on the basis of eqn (S1)–(S3) (ESI),†^24,25^ where the relevant parameters were explained in detail.

## 3. Results and discussion

### 3.1. Purification of FGD gypsum

**Fig. 1:**
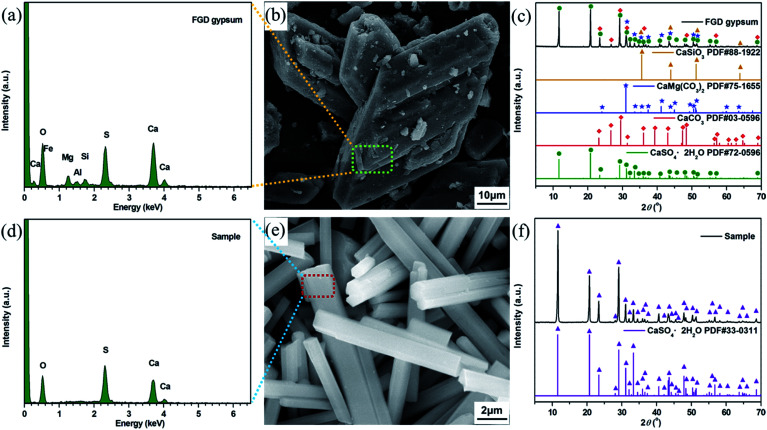
(a) EDX pattern, (b) SEM image and (c) PXRD patterns of the FGD gypsum; (d) EDX spectrum, (e) SEM image and (f) PXRD pattern of the purified FGD gypsum.

The FGD gypsum obtained from Zhongwei power plant could not be directly used for the preparation of composite materials before purification, owing to the coexistence of large amounts of impurities. As illustrated in Fig. 1b, it consists mostly of block-shaped particles with very rough surfaces. The well-resolved PXRD peaks depicted in Fig. 1c can be indexed to those of monoclinic CaSO_4_·2H_2_O (JCPDS 72-0596), rhombohedral CaCO_3_ (JCPDS 03-0596), rhombohedral CaMg(CO_3_)_2_ (JCPDS 75-1655) and cubic CaSiO_3_ (JCPDS 88-1922), respectively. Besides, the presence of trace amounts of Al, F, Fe, K, Na, Cl and P elements in FGD gypsum can be confirmed by EDX and XRF analyses (Fig. 1a, Tables S1 and S2 (ESI)†). When treated with HCl solution under high-temperature and high-pressure conditions, the block-shaped particles were gradually dissolved, giving rise to the formation of a pale yellow solution. After filtration, rod-like particles with relatively smooth surface (Fig. 1e) were eventually precipitated with the addition of EtOH. The corresponding PXRD pattern in Fig. 1f exhibits strong and sharp diffraction peaks, indicating that the purified sample has very high crystallinity. There is no obvious evidence for impurity phases, and all peak positions are perfectly in accordance with those of monoclinic CaSO_4_·2H_2_O (JCPDS 33-0311). In addition, only O, S and Ca elements could be detected in EDX analysis (Fig. 1d and Table S3 (ESI)†), and the corresponding atomic ratio was estimated to be 6 : 1:1, further suggesting that a single-crystalline phase of CaSO_4_·2H_2_O was formed.

### 3.2. Synthesis of MoO_2_/CaSO_4_ composites

**Fig. 2:**
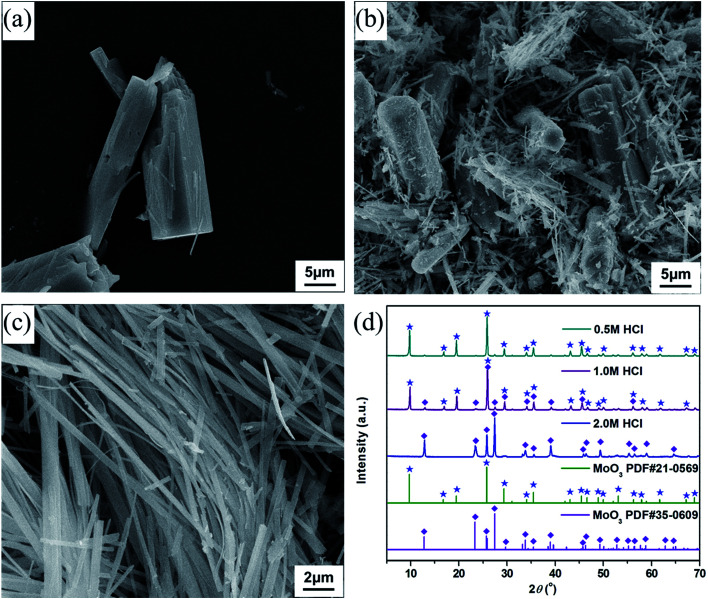
SEM images of the samples derived from HM by acidification with (a) 0.5 M, (b) 1.0 M and (c) 2.0 M HCl; (d) PXRD patterns of the acidified products formed in HCl solution with different concentrations.

To synthesize molybdenum oxides, HM was acidified with different HCl solutions by a hydrothermal process at 180 °C. As shown in Fig. 2a, samples with a columnar structure were obtained when the HCl concentration was 0.5 M. The PXRD pattern displayed in Fig. 2d can be well indexed as the pure phase of hexagonal MoO_3_ (h-MoO_3_, JCPDS 21-0596) with the calculated lattice constants of *a* = 10.618 Å and *c* = 14.652 Å. By increasing the HCl concentration from 0.5 to 2.0 M, the columnar structure began to break down and disappeared gradually, whereas a strip-shaped structure (Fig. 2b and c) emerged simultaneously. All diffraction peaks of the strip-shaped samples (Fig. 2d) could be consistent with those of orthorhombic MoO_3_ (α-MoO_3_, JCPDS 35-0609) with the calculated lattice parameters of *a* = 3.927 Å, *b* = 13.624 Å and *c* = 3.679 Å. This result indicates that the HCl concentration has significant impacts on the morphology and phase structure of MoO_3_, and the excess HCl as an acidic catalyst can trigger and promote its structural phase transition from h-MoO_3_ to α-MoO_3_. Similar results were also observed in previous reports.^26,27^ However, neither the metastable h-MoO_3_ nor the thermodynamically stable α-MoO_3_ formed here could be attached to the surface of the as-prepared CaSO_4_·2H_2_O to form composite materials, because of their relatively large particle sizes.

**Fig. 3:**
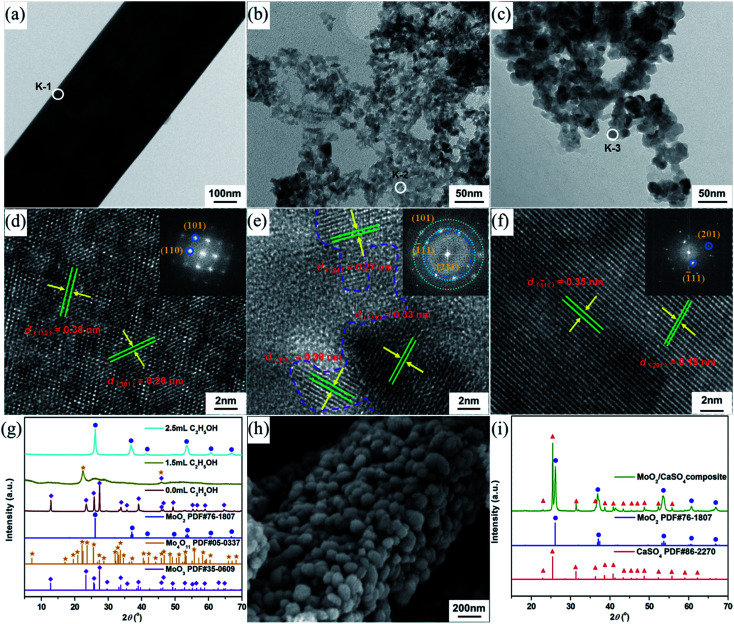
TEM images of the samples derived from HM in the mixed solvent containing 15 mL of 3.0 M HCl and (a) 0.0 mL, (b) 1.5 mL and (c) 2.5 mL of EtOH; HR-TEM images of the selected area (d) K-1, (e) K-2 and (f) K-3 separately marked in (a–c) (insets: FFT patterns); (g) PXRD patterns of the products obtained in mixed solvent systems with various solvent ratios; (h) SEM image and (i) PXRD pattern of the MoO_2_/CaSO_4_ composite.

Generally speaking, alcohols can serve as solvents and reductants, as well as the stabilizers of grain growth and hydration processes.^28,29^ Therefore, EtOH was added to the HCl solutions in order to get smaller MoO_2_ particles than the as-prepared MoO_3_ particles. As indicated in Fig. 3a, samples with a strip-shaped structure were formed without the addition of EtOH. The clear lattice fringes in the high-resolution TEM (HR-TEM) image and the symmetrical diffraction spots in the fast Fourier transform (FFT) pattern (Fig. 3d) revealed that the strip-shaped sample had a high crystallinity and exhibited a single-crystalline structure. The corresponding interplanar spacings (0.38 and 0.26 nm) were in accord with those of the (110) and (101) planes of α-MoO_3_ (JCPDS 35-0609), respectively. Moreover, the PXRD pattern (Fig. 3g) could also be in good agreement with that of α-MoO_3_, which was consistent with the result of the HR-TEM analysis. With the addition of EtOH, the morphology of samples changed significantly. The strip-shaped structure was disappeared, and small nanoparticles were finally formed (Fig. 3b and c). As shown in Fig. 3e, when the dosage of EtOH was 1.5 mL, the measured interplanar spacings (0.27, 0.39 and 0.33 nm) of the sample could be assigned to the (101) plane of α-MoO_3_, the (211) plan of Mo_4_O_11_ (JCPDS 05-0337) and the (1̄11) plane of MoO_2_ (JCPDS 76-1807), respectively, and its FFT pattern derived from the HR-TEM image presented indistinct diffraction rings, implying a relatively poor crystallinity. Nevertheless, the mixed-phase nanoparticles were then transformed into single-phase nanoparticles when the dosage of EtOH was increased to 2.5 mL. The measured interplanar spacings (0.19 and 0.35 nm, Fig. 3f) of the sample could be separately indexed to the (201) and (1̄11) planes of MoO_2_, and its FFT pattern derived from the HR-TEM image displayed regular diffraction spots, suggesting a relatively high crystallinity and a single-crystalline phase. Similar results were further confirmed by PXRD analyses (Fig. 3g). Additionally, the morphology and structure of the formed samples were no longer changeable when the EtOH volume was more than 2.5 mL. In an aqueous HCl solution, gypsum is liable to undergo the phase transformation from CaSO_4_·2H_2_O to CaSO_4_ over a wide temperature range by reason of the decrease in water activity.^30^ As a consequence, the lattice water molecules of the as-prepared CaSO_4_·2H_2_O were eventually lost, and a rod-shaped MoO_2_/CaSO_4_ composite with a significant amount of MoO_2_ nanoparticles assembled to the outer surface of CaSO_4_ (Fig. 3h) was formed during the mixed solvothermal process. The structure and elemental composition of this composite material were further verified by PXRD and EDX analyses, respectively (Fig. 3i and S1 (ESI)†).

**Fig. 4:**
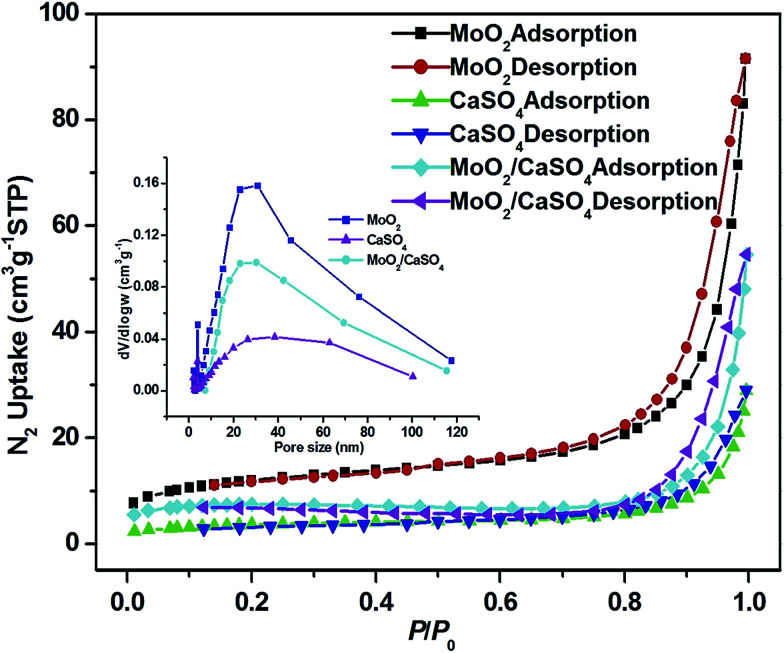
Nitrogen adsorption–desorption isotherms of MoO_2_, CaSO_4_ and MoO_2_/CaSO_4_ composites (inset: pore size distribution curves).

In order to investigate the textural characteristics of the as-formed samples, or rather their specific surface areas and pore size distributions, the N_2_ adsorption–desorption data of them were collected. As depicted in Fig. 4, all isotherms can be categorized as type II, with the type H3 hysteresis loops observed in the range of 0.7–1.0 *P*/*P*_0_. This result suggests that they are non-porous solids, and non-structural mesopores have been formed *via* particle packing,^31,32^ which can be further validated by the broad Barrett–Joyner–Halenda (BJH) pore size distribution curves (inset of Fig. 4) derived from the desorption profiles of isotherms. The measured BET specific areas of MoO_2_, CaSO_4_ and MoO_2_/CaSO_4_ composites are 42.6, 13.0 and 25.4 m^2^ g^−1^, respectively. Apparently, the specific area of this MoO_2_/CaSO_4_ composite becomes larger than that of CaSO_4_ as the deposition of MoO_2_ particles.

### 3.3. Adsorption of synthetic dyes on MoO_2_/CaSO_4_ composites

**Fig. 5:**
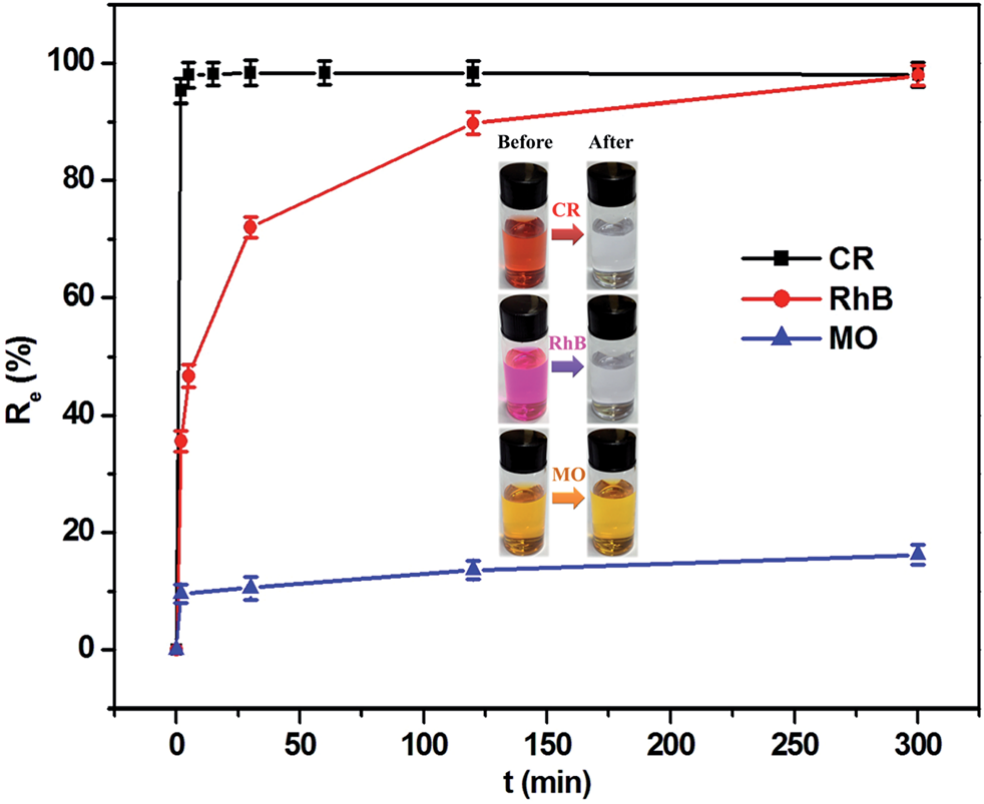
Removal efficiencies of the synthetic dyestuffs in aqueous solution (initial concentrations: 250 mg L^−1^ for CR, 20 mg L^−1^ for RhB and 10 mg L^−1^ for MO) under the action of the MoO_2_/CaSO_4_ composite at different contact time (inset: color variation of the dye solution before and after adsorption at 303.5 K).

The capability of this composite as an adsorbent in removing organic dye contaminants from aqueous solutions was investigated at 303.5 K. As can be seen from Fig. 5, the adsorption equilibrium was achieved within a short time period. For CR dye, its removal rate reached 98.0% in the initial 5 min, which was equivalent to the adsorption quantity of 122.5 mg g^−1^. The RhB removal efficiency achieved 89.8% when the period of time was 120 min, corresponding to the adsorption capacity of 9.0 mg g^−1^. However, the removal efficiency of MO was only approximately 16.2% even if the adsorption time was extended to 300 min, in which the adsorption capacity corresponded to 0.8 mg g^−1^. This result implies that the MoO_2_/CaSO_4_ composite has an excellent adsorption capability towards CR and RhB. As sulfonated anionic dyes, CR and MO belong to the category of Lewis bases, but RhB as one of the non-sulfonated cationic dyes can be assigned to the classification of Lewis acids. In our and others' previous studies,^33,34^ gypsum and its subhydrates have been found to possess the capacity to interact with anionic dyes. Moreover, surface hydroxylation tends to occur when molybdenum oxides are exposed to water, which is beneficial to the adsorption of cationic dyes.^23^ Consequently, this MoO_2_/CaSO_4_ composite can theoretically bind with cationic and anionic dyes *via* electrostatic attraction. Nevertheless, the extremely low adsorption ratio of MO might be attributed to its stereochemical structure and the lack of effective functional groups.

### 3.4. Effects of adsorbent dosage and solution pH on dye removal

**Fig. 6:**
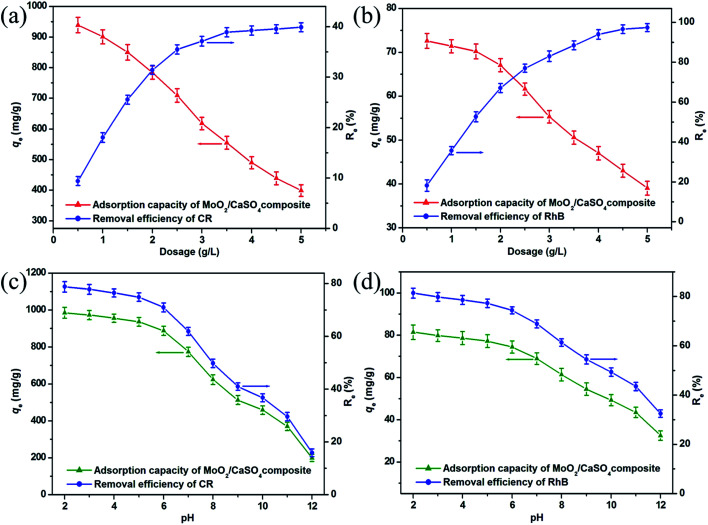
Effect of adsorbent dosage on the adsorption processes of (a) CR and (b) RhB onto the MoO_2_/CaSO_4_ composite at 303.5 K; effect of solution pH on the adsorption processes of (c) CR and (d) RhB onto the MoO_2_/CaSO_4_ composite at 303.5 K.

To estimate the influence of adsorbent dose on dye adsorption onto this MoO_2_/CaSO_4_ composite, removal experiments were performed over a dosage range between 0.5 and 5.0 g L^−1^. As shown in Fig. 6a and b, similar trends were found in the removal efficiencies and adsorption quantities of CR and RhB, respectively. Initially, prominent increases in removal ratios from 9.4% to 39.9% for CR and 18.2% to 97.4% for RhB were observed with the increasing adsorbent dose from 0.5 to 4.0 g L^−1^, indicating the emergence of more and more active adsorption sites. Subsequently, a leveling off was achieved with further increases in adsorbent dosage, which might be attributed to the partial aggregation of adsorbent particles. However, the equilibrium adsorption quantities of the dyes per unit mass notably decreased from 939.5 to 398.8 mg g^−1^ for CR and 72.6 to 39.1 mg g^−1^ for RhB as the adsorbent dose increased, which might be correlated with the limited availability of dye molecules giving rise to the unsaturation of binding sites. Considering both the removal efficiency and adsorption capacity, we ultimately selected a dosage of 2.0 g L^−1^ for further research.

Taking into account the essential role of solution pH in dye removal processes, its effect on dye adsorption onto this MoO_2_/CaSO_4_ composite was also investigated. As exhibited in Fig. 6c and d, similar trends in the removal efficiencies and adsorption quantities of CR and RhB were separately noted. With the increase of initial pH from 2.0 to 12.0, the removal efficiencies of CR and RhB decreased from 78.8% to 15.9% and 81.4% to 32.5%, respectively. Meanwhile, obvious downtrends in adsorption quantities from 985.2 to 199.5 mg g^−1^ for CR and 81.4 to 32.5 mg g^−1^ for RhB were also observed, respectively. These results can be well explained by the change in surface charges of samples. As one of the acid (anionic) dyes, CR has an isoelectric point around 3.0.^35^ As for RhB, it is a basic (cationic) dye in which the p*K*_a_ value of the aromatic carboxyl group is near 3.1.^36^ When the solution pH is increased from 2.0 to 12.0, both dyes will undergo a transformation from their cationic states to anionic states. In addition, the zeta potential values of this MoO_2_/CaSO_4_ composite are negative throughout the pH range (Fig. S2, ESI†). Therefore, the high adsorption capability at low pH can be ascribed to electrostatic attraction. When the adsorption process takes place under strong alkaline conditions, all dye molecules are negatively charged, and the surface hydroxylation of this MoO_2_/CaSO_4_ composite is enhanced. Thus, electrostatic repulsion eventually leads to the decrease of the adsorption capacity. It should be noted that the relatively weak adsorptive capacity of this MoO_2_/CaSO_4_ composite at high pH might also be correlated with the synergistic effect of hydrogen bonds and van der Waals interactions. Considering the environmental damage caused by the solution acidity, we finally chose a pH of 7 for further research.

### 3.5. Adsorption isotherms

To research the adsorption behavior of this MoO_2_/CaSO_4_ composite towards CR and RhB in detail, four common isotherm models including the Langmuir, Freundlich, Dubinin–Radushkevich (D–R) and Temkin models were employed in this study to investigate the adsorption equilibrium process.

**Fig. 7:**
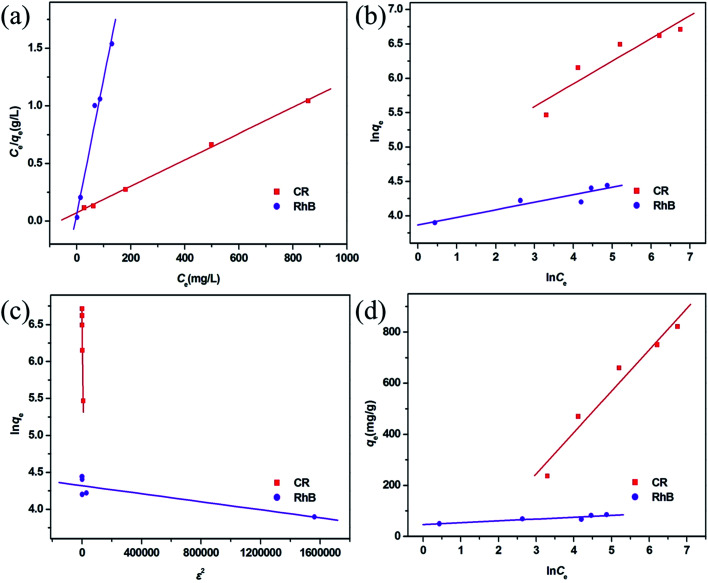
Adsorption isotherms fitted by (a) Langmuir, (b) Freundlich, (c) D–L and (d) Temkin models for CR and RhB adsorption.

In the Langmuir model, interactions among adsorbate molecules are excluded from consideration, and a monolayer adsorption process is assumed to take place on the homogeneous surface of an adsorbent. As shown in eqn (S4) (ESI),† the ratio of the equilibrium concentration (*C*_e_, mg L^−1^) to the equilibrium adsorption capacity (*q*_e_, mg g^−1^) for an adsorbate in solution can be calculated according to the relationship of a function (*k*_L_, L mg^−1^) associated with the adsorption free energy and its maximum adsorbed quantity (*q*_max_, mg g^−1^).^37^ In addition to the correlation coefficient (*R*^2^), another parameter for evaluating the applicability of the Langmuir model is the separation factor (*R*_L_), which is a dimensionless constant and can be represented by eqn (S5) (ESI).†^33^ For *R*_L_ = 0, the adsorption process is non-reversible. For 0 < *R*_L_ < 1, the Langmuir model is appropriate for describing adsorption processes. For *R*_L_ = 1, the adsorption process is linear. For *R*_L_ > 1, the Langmuir model is unsuitable. As shown in Fig. 7a, both scatter plots are well consistent with the Langmuir model. Moreover, the corresponding *R*^2^ values are greater than 0.99, and all *R*_L_ values are in the range between 0.022 and 0.143 (Table S4, ESI†). This result reveals that the adsorption of CR and RhB onto this MoO_2_/CaSO_4_ composite belongs to monolayer adsorption processes.

In the Freundlich model, it is assumed that a multilayer adsorption process occurs on the non-homogeneous surface of an adsorbent. It can be expressed by eqn (S6) (ESI),†^23^ where the relevant parameters are defined in detail. As illustrated in Fig. 7b, both scatter diagrams are inconsistent with the Freundlich model. Besides, the corresponding *R*^2^ values are less than 0.86 (Table S4, ESI†), which indicates that the adsorption of CR and RhB onto this MoO_2_/CaSO_4_ composite cannot be classified as multilayer adsorption processes.

The D–R model can be applied to estimate whether an adsorption process is physical or chemical. It is usually represented by eqn (S7) (ESI)† and described by the relationship of the Polanyi potential *ε* given by eqn (S8) (ESI),† the maximum adsorbed quantity (*q*_m_, mg g^−1^), and a function (*k*_D_, mol^2^ kJ^−2^) which is related to the mean adsorption free energy (*E*, kJ mol^−1^) calculated on the basis of eqn (S9) (ESI).†^38^ The adsorption isotherms fitted by this model for CR and RhB are depicted in Fig. 7c, and their relevant parameters are listed in Table S4 (ESI).† Both *R*^2^ values are less than 0.90, suggesting that it is inappropriate to describe the adsorption processes of CR and RhB onto this MoO_2_/CaSO_4_ composite by the D–R model.

In the Temkin model, interactions among adsorbate molecules are taken into account, and the adsorption heat of all molecules is considered to be linearly decreased with the increasing adsorption quantity. As expressed in eqn (S10) (ESI),†*q*_e_ is calculated according to the relationship between the Temkin isotherm constant (*k*_T_, L mg^−1^) and the adsorption heat (*b*, equivalent to −Δ*H*, kJ mol^−1^).^39^ The adsorption isotherms fitted by this model for CR and RhB are displayed in Fig. 7d. It can be observed that the scatter diagram of CR is more consistent with the Temkin model than that of RhB. A similar result can also be obtained by the values of *R*^2^ (Table S4, ESI†). In addition, the value of *b* for CR is positive, implying that its adsorption onto this MoO_2_/CaSO_4_ composite is an exothermic process.

By comparing the *R*^2^ values of the four isotherm models, it can be found that the adsorption equilibrium processes of CR and RhB can be best described by the Langmuir isotherm model. The corresponding *q*_max_ values of them adsorbed onto this MoO_2_/CaSO_4_ composite are 853.54 mg g^−1^ and 86.38 mg g^−1^ (Table S4, ESI†), respectively. Moreover, as shown in Table S5 (ESI),† this MoO_2_/CaSO_4_ composite displays a more excellent adsorption capability than some other reported adsorbents.^23,40–44^ Therefore, it can be used as a potential candidate for water purification.

### 3.6. Thermodynamic studies

To get insight into the influence of temperature on dye adsorption onto this MoO_2_/CaSO_4_ composite, three thermodynamic parameters including the Gibbs free energy change (Δ*G*^0^, kJ mol^−1^), enthalpy change (Δ*H*^0^, kJ mol^−1^) and entropy change (Δ*S*^0^, J mol^−1^ K^−1^) were calculated. Their equations can be separately expressed by eqn (S11) and (S12) (ESI),†^41^ where Δ*G*^0^ is described in terms of the distribution coefficient (*K*_q_, L g^−1^) of an adsorbent, and ln *K*_q_ is described according to the relationship of *T*, Δ*H*^0^ and Δ*S*^0^. All values of the corresponding thermodynamic parameters are shown in Table S6 (ESI).† It can be found that all values of Δ*G*^0^ for CR and RhB are negative, which means that their adsorption processes are spontaneous. The values of Δ*H*^0^ and Δ*S*^0^ for CR are negative, implying that its adsorption on this MoO_2_/CaSO_4_ composite is exothermic, and there is a decrease in randomness at the solid/liquid interface during the adsorption process. Conversely, the values of Δ*H*^0^ and Δ*S*^0^ for RhB are positive. This result suggests that the adsorption of RhB is endothermic, and there is an increase in randomness at the solid/liquid interface during the adsorption process.

### 3.7. Adsorption kinetics

To better understand the rate-controlling step and adsorption mechanism of this MoO_2_/CaSO_4_ composite towards CR and RhB, four common kinetic models including the pseudo-first-order, pseudo-second-order, Elovich and intra-particle diffusion models were utilized in this study to investigate the relationship of the adsorption quantity and the contact time.

**Fig. 8:**
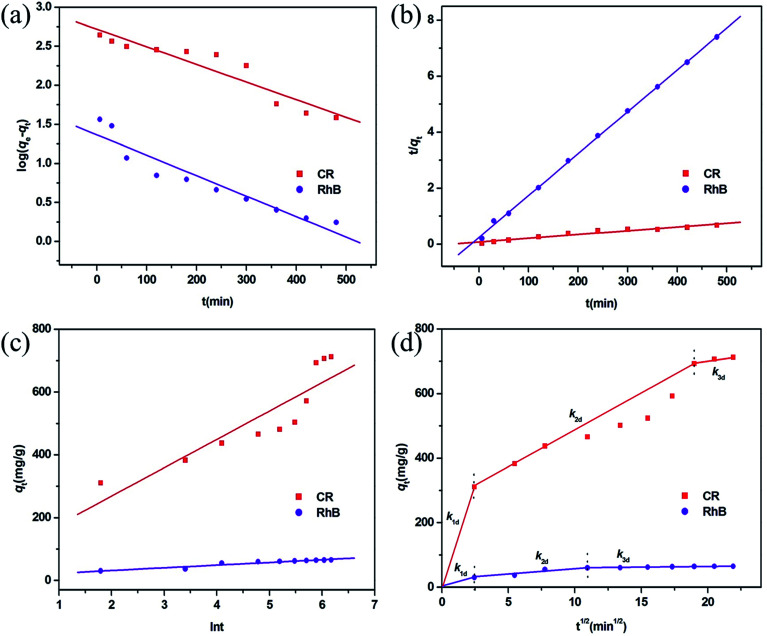
Adsorption kinetic plots fitted by (a) pseudo-first-order, (b) pseudo-second-order, (c) Elovich, and (d) intra-particle diffusion models for CR and RhB adsorption.

The pseudo-first-order kinetic model is given by eqn (S13) (ESI),†^44^ where the relevant parameters are elaborated. As illustrated in Fig. 8a, both scatter diagrams are inconsistent with this linear model. Moreover, the corresponding values of *R*^2^ are less than 0.91, and their calculated values of *q*_e_ do not match with the experimental values (Table S7, ESI†). These results imply that it is unsuitable to describe the adsorption processes of CR and RhB by the pseudo-first-order model.

The pseudo-second-order sorption model can be represented by eqn (S14) (ESI),†^45^ where the ratio *t*/*q*_e_ can be calculated according to the relationship of time (*t*, min) and a rate constant (*k*_2_, g mg^−1^ min^−1^). As demonstrated in Fig. 8b, both scatter plots coincide better with this linear model. Additionally, their corresponding values of *R*^2^ are greater than 0.95, and the calculated values of *q*_e_ are in close proximity to the experimental values (Table S7, ESI†). These results suggest that the adsorption processes of CR and RhB onto this MoO_2_/CaSO_4_ composite can be well described by the pseudo-second-order rate equation.

The Elovich kinetic model is given by eqn (S15) (ESI),†^43^ where *q*_*t*_ can be calculated based on the relationship between the Elovich desorption constant (*β*, g mg^−1^) and the initial adsorption rate (*α*, mg g^−1^ min^−1^). As displayed in Fig. 8c, most of the experimental data do not meet this linear equation. In addition, the corresponding values of *R*^2^ are less than 0.93 (Table S7, ESI†), which means that the adsorption processes of CR and RhB cannot be described by this kinetic model.

The intra-particle diffusion model can usually be applied to evaluate the effect of molecular diffusion on an adsorption process. As expressed in eqn (S16) (ESI),†^44^*q*_*t*_ can be calculated according to the relationship between a constant *C*_*i*_, whose value is correlated with the boundary layer thickness of molecular diffusion, and the rate constant of intra-particle diffusion at stage *i* (*k*_id_, mg g^−1^ min^−1/2^). Fig. 8d demonstrates that all scatter plots are nonlinear and can be split into three linear parts, which implies that the adsorption processes of CR and RhB are multi-step processes. Initially, there is a significant increase of the adsorption quantity in the first linear portion, which can be ascribed to the surface adsorption caused by the boundary layer effect. Subsequently, the removal efficiency is dominated by intra-particle diffusion, and the adsorption rate in the second linear portion is lower than that in the first linear part. Finally, the adsorption rate reaches the lowest value, and the adsorption equilibrium is achieved in the third linear region. As shown in Table S7 (ESI),† all values of *k*_id_ for CR are larger than those for RhB. Moreover, the values of *k*_id_ for each dye present a declining trend during their adsorption processes, which is consistent with the characteristic of the multi-step adsorption process. These results suggest that both surface adsorption and intra-particle diffusion influence the adsorption processes of CR and RhB onto the obtained MoO_2_/CaSO_4_ composite.

### 3.8. ATR-FT-IR spectral analysis

**Fig. 9:**
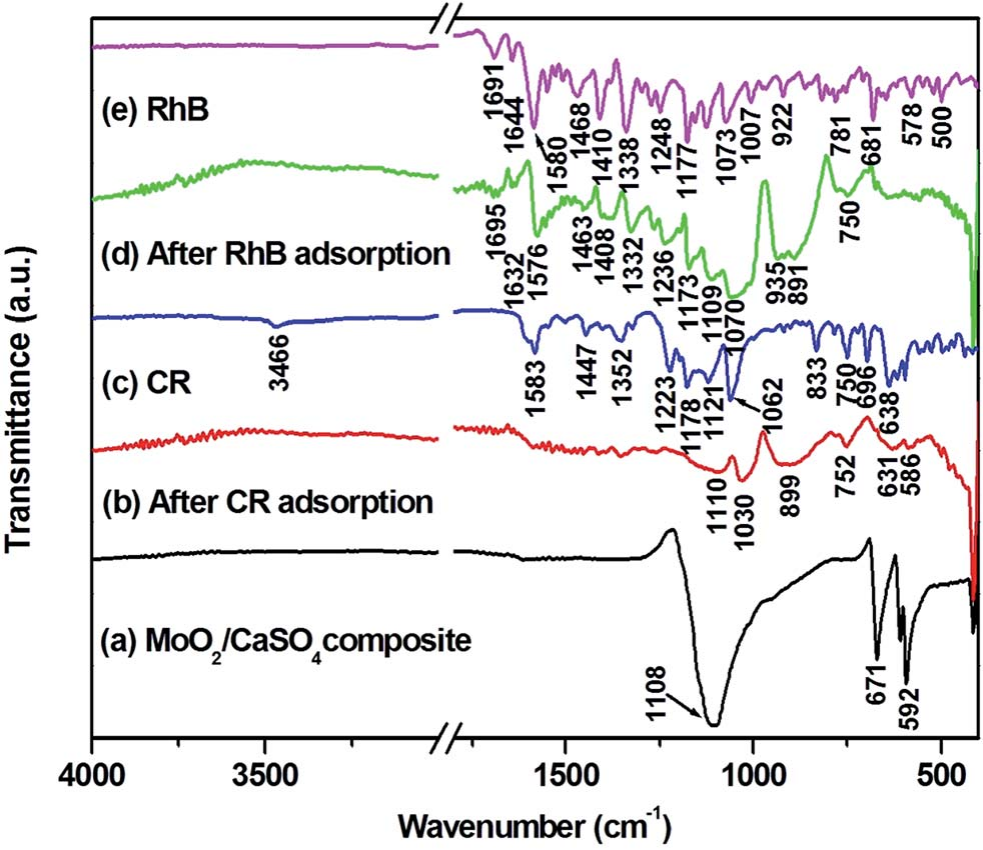
ATR-FT-IR spectra of (a) MoO_2_/CaSO_4_ composites, (b) MoO_2_/CaSO_4_ composites after CR adsorption, (c) CR, (d) MoO_2_/CaSO_4_ composites after RhB adsorption and (e) RhB dye.

To gain more details about interactions between adsorbents and adsorbates, the ATR-FT-IR spectra of CR, RhB and MoO_2_/CaSO_4_ composites before and after adsorption were analyzed. As exhibited in Fig. 9, the characteristic vibration bands for CR and RhB (Fig. 9c and e) are in accord with those in previous reports,^23,46^ and all absorption bands of the MoO_2_/CaSO_4_ composite after CR and RhB adsorption (Fig. 9b and d) become relatively weaker than those before adsorption (Fig. 9a). For CR adsorption, most of the characteristic peaks for CR are absent owing to the confinement effect caused by the adsorbent.^47^ Besides, the peak of the –SO_3_^−^*ν*_1_ stretching vibration (1062 cm^−1^) shifts to a lower wavenumber (1030 cm^−1^), which is an obvious evidence for the interaction between CR and the MoO_2_/CaSO_4_ composite. For RhB adsorption, many peaks of RhB appear in the corresponding spectrum, including the peaks at 1695 cm^−1^ for the C=O stretching vibration, in the range of 1576–1463 cm^−1^ for aromatic ring vibrations, at 1408 cm^−1^ for the bending vibration of CH_2_ in =N^+^(C_2_H_5_)_2_, at 1332 cm^−1^ for the stretching vibration of the C–N-linked benzene ring, at 1173 cm^−1^ for the asymmetric stretching vibration of C–O–C and 1070 cm^−1^ for the C–OH stretching vibration. Additionally, the peaks at 1644 cm^−1^ for the bending vibration of C=N^+^ and 1248 cm^−1^ for the stretching vibration of C–N in =N^+^(C_2_H_5_)_2_ also shift to lower wavenumbers (1632 and 1236 cm^−1^), respectively. These findings suggest that chemical adsorption plays an important role in the adsorption of CR and RhB onto this MoO_2_/CaSO_4_ composite.

### 3.9. XPS spectral analysis

**Fig. 10:**
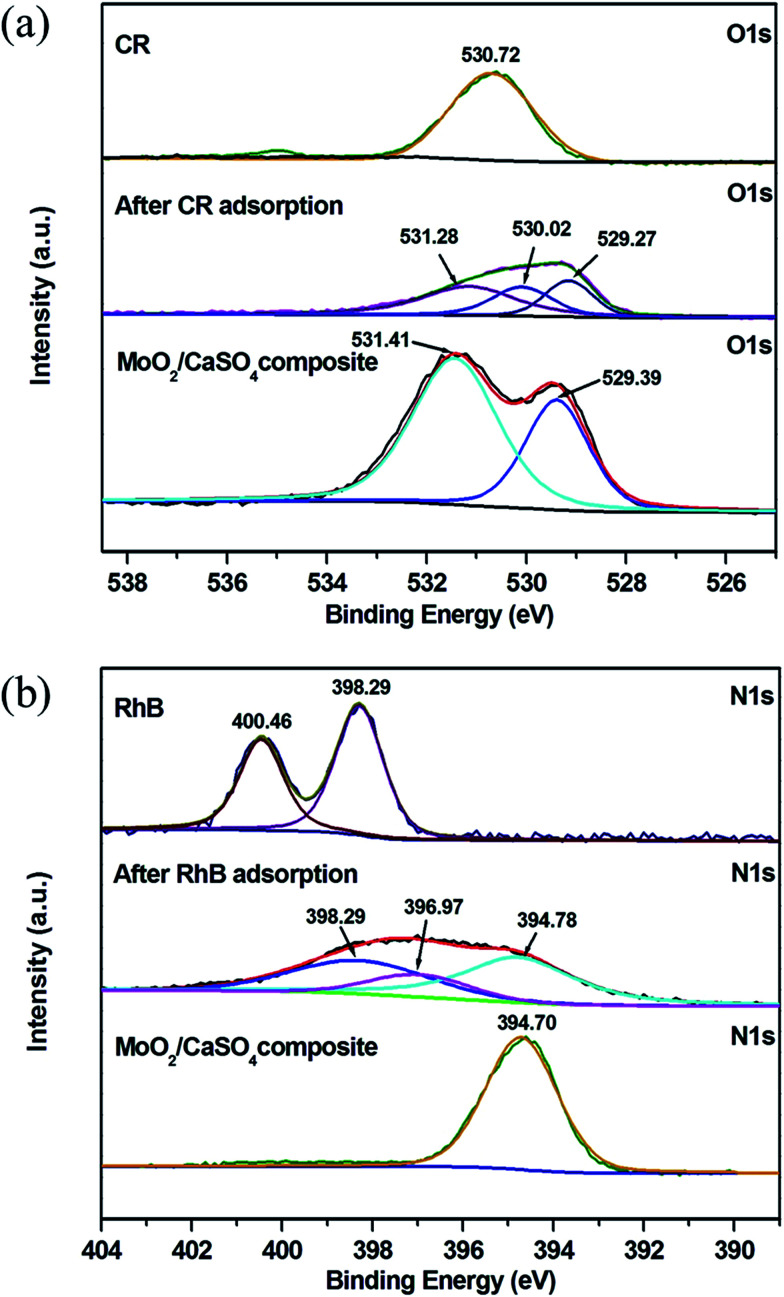
(a) High-resolution O 1s scan XPS spectra of MoO_2_/CaSO_4_ composites, MoO_2_/CaSO_4_ composites after CR adsorption and CR, (b) high-resolution N 1s scan XPS spectra of MoO_2_/CaSO_4_ composites, MoO_2_/CaSO_4_ composites after RhB adsorption and RhB dye.

To get further evidence for the findings obtained from the ATR-FT-IR spectral analysis, XPS measurements were also conducted. As presented in Fig. S3 (ESI),† the elementary C, O, Mo, Ca and S can be observed in the full scan spectrum of the MoO_2_/CaSO_4_ composite before and after adsorption. The existence of C in the pure MoO_2_/CaSO_4_ composite might be related to the adsorbed CO_2_ or adventitious hydrocarbons.^48^ Besides, N is also detected after dye adsorption, which is a characteristic element of CR and RhB. This result signifies that CR and RhB have been adsorbed by the MoO_2_/CaSO_4_ composite. As displayed in Fig. 10a, the O 1s peak for –SO_3_^−^ in CR molecules appears at 530.72 eV, whilst the O 1s peaks for SO_4_^2−^ and Mo=O in the pure MoO_2_/CaSO_4_ composite appear at 531.41 and 529.39 eV, respectively. After CR adsorption, the O 1s region of the sample can be fitted into three peaks: the peaks at 531.28 and 529.27 eV are in accordance with those of SO_4_^2−^ and Mo=O in the MoO_2_/CaSO_4_ composite, and the peak at 530.02 eV can be ascribed to that of oxygen related bonds between the MoO_2_/CaSO_4_ composite and CR molecules. As shown in Fig. 10b, the N 1s peaks for =N^+^(C_2_H_5_)_2_ and –N(C_2_H_5_)_2_ in RhB molecules are located at 400.46 and 398.29 eV, respectively. The peak at 394.70 eV for the pure MoO_2_/CaSO_4_ composite can be attributed to the Mo 3p_3/2_ peak. After RhB adsorption, it can be found that the N 1s region of the sample can also be fitted into three peaks: the peak at 394.78 eV is well matched with the Mo 3p_3/2_ peak of the MoO_2_/CaSO_4_ composite, the peak at 398.29 eV is consistent with that of –N(C_2_H_5_)_2_ in RhB molecules, and the peak at 396.97 eV can be assigned to that of nitrogen related bonds between the MoO_2_/CaSO_4_ composite and RhB molecules. These results imply that chemisorption is the main interaction between the dyes and the MoO_2_/CaSO_4_ composite.

## 4. Conclusions

In summary, a kind of rod-shaped MoO_2_/CaSO_4_ composite, in which CaSO_4_ matrices are decorated with MoO_2_ nanoparticles, has been synthesized by using HM and FGD gypsum *via* a mixed-solvothermal approach. Moreover, it displays an excellent adsorption capability towards anionic CR and cationic RhB dyes. The adsorption quantities of the two dyes per unit mass notably decrease as solution pH and adsorbent dose increase. However, their removal efficiencies are improved by increasing adsorbent dosage. The adsorption equilibrium data are best fitted by the Langmuir model, and the calculated maximum adsorption quantities at 303.5 K are 853.54 mg g^−1^ for CR and 86.38 mg g^−1^ for RhB, respectively, which are superior to other common adsorbents. Their adsorption kinetic data can be well matched with the pseudo-second-order model. Additionally, the thermodynamic analysis demonstrates that the CR adsorption is an exothermic process, while the RhB adsorption is an endothermic process. Both of them are multi-step chemisorption processes influenced by surface adsorption and intra-particle diffusion. The MoO_2_/CaSO_4_ composite has great potentials as an alternative adsorbent for the purification of the wastewaters contaminated by synthetic dyes.

## Conflicts of interest

There are no conflicts of interest to declare.

## Supplementary Material

RA-008-C7RA11292K-s001
