# Supplementary material for: Facile synthesis of MoO2/CaSO4 composites as highly efficient adsorbents for congo red and rhodamine B
Source: RSC Adv. 2018 Jan 4;8(3):1621–31. doi: 10.1039/c7ra11292k (PMC9077254; doi:10.1039/c7ra11292k)
Supplement: RA-008-C7RA11292K-s001 [file RA-008-C7RA11292K-s001.pdf]

Electronic Supplementary Material (ESI) for RSC Advances.
This journal is © The Royal Society of Chemistry 2017

Electronic Supplementary Information (ESI)

Facile synthesis of $MoO_2/CaSO_4$ composites as highly efficient adsorbents for Congo red and Rhodamine B

Xin-Jian Jia, Jinshu Wang,* Junshu Wu, Weili Teng, Bingxin Zhao, Hongyi Li and Yucheng Du

Key Laboratory of Advanced Functional Materials for Ministry of Education, College of Materials Science and Engineering, Beijing University of Technology, Beijing 100124, China. E-mail: wangjsh@bjut.edu.cn

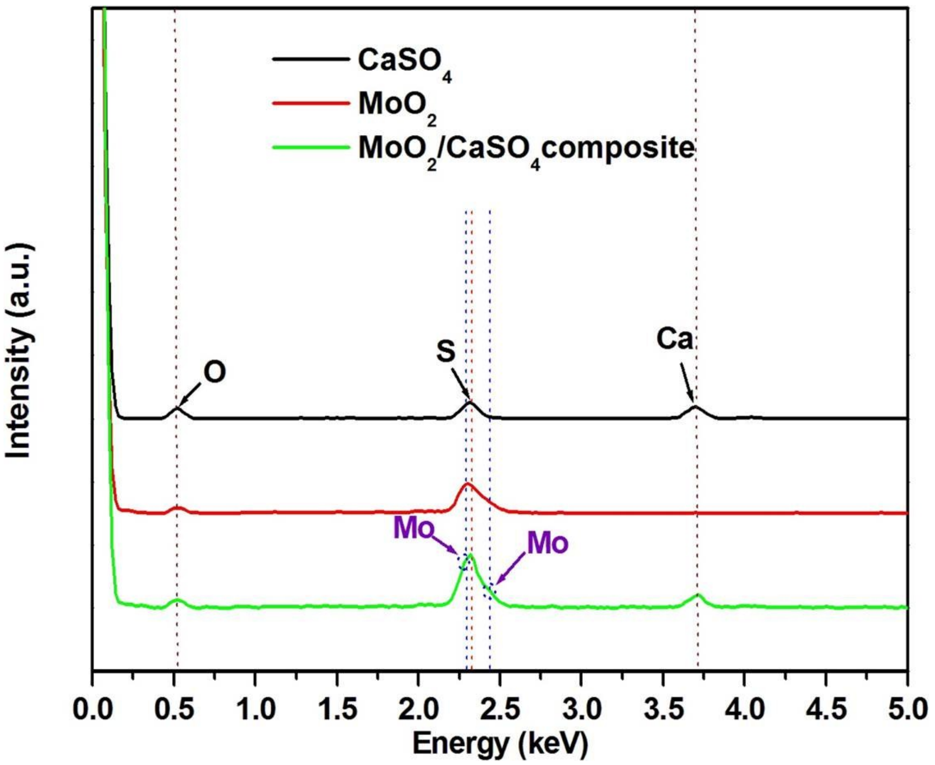

Fig. S1. EDX patterns of $CaSO_4$, MoO_2 and $MoO_2/CaSO_4$ composites.

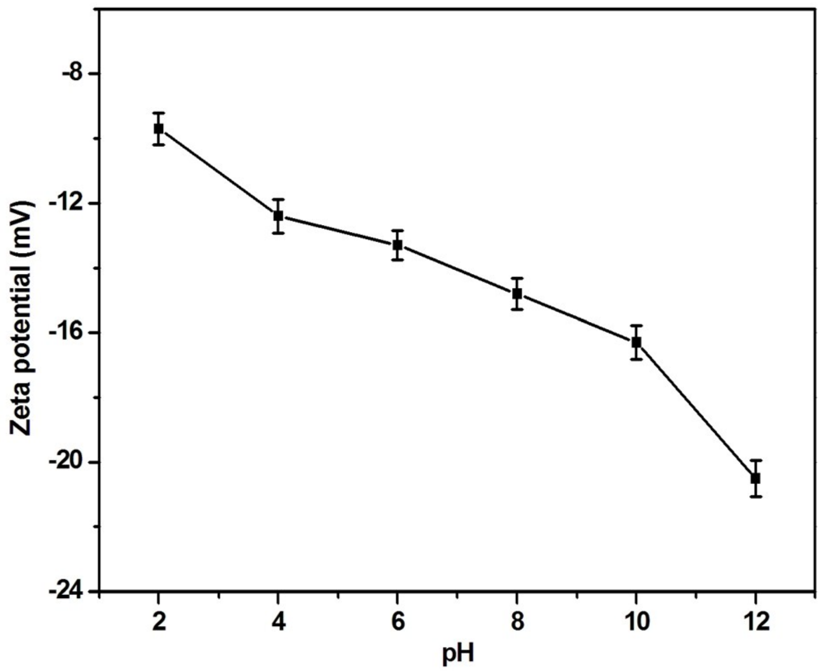

Fig. S2 Zeta potential values of the $MoO_2/CaSO_4$ composite over a pH range between 2.0 and 12.0.

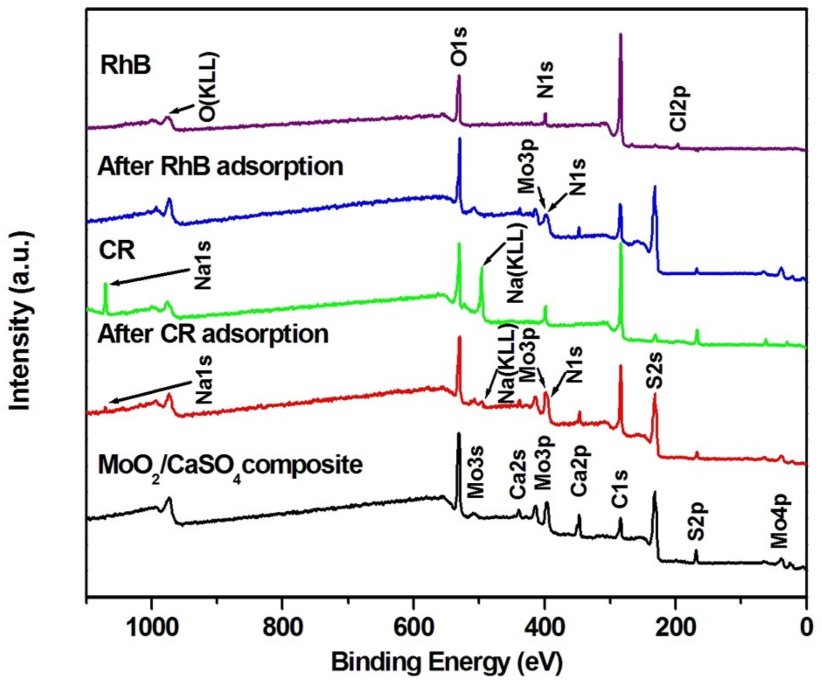

Fig. S3. XPS survey scan spectra of $MoO_2/CaSO_4$ composites, $MoO_2/CaSO_4$ composites after CR adsorption, CR, $MoO_2/CaSO_4$ composites after RhB adsorption and RhB.

Eq. S1. Equation for the removal efficiency (R_e) of a dye onto an adsorbent.

$$R_e = \frac{C_0 - C_t}{C_0} \times 100\%$$

C_0 (mg·L^{-1}) is the initial concentration of a dye solution; C_t (mg·L^{-1}) is the dye concentration at time t.

Eq. S2. Equation for the adsorption capacity (q_e, mg·g^{-1}) of a dye onto an adsorbent at equilibrium.

$$q_e = \frac{C_0 - C_e}{m} V$$

C_e (mg·L^{-1}) is the equilibrium concentration of a dye solution; m (g) is the mass of an adsorbent; and V (L) is the volume of a dye solution.

Eq. S3. Equation for the adsorption quantity (q_t, mg·g^{-1}) of a dye onto an adsorbent at time t.

$$q_t = \frac{C_0 - C_t}{m} V$$

Eq. S4. Equation for the Langmuir model.

$$\frac{C_e}{q_e} = \frac{C_e}{q_{max}} + \frac{1}{q_{max} k_L}$$

In this model, q_{max} (mg·g^{-1}) and k_L (L·mg^{-1}) are Langmuir isotherm constants separately representing the maximum adsorbed quantity and a function associated with the adsorption free energy.

Eq. S5. Equation for the separation factor R_L.

$$R_L = \frac{1}{1 + k_L C_0}$$

Eq. S6. Equation for the Freundlich model.

$$\ln q_e = \ln k_F + \frac{1}{n}\ln C_e$$

In this model, k_F ((mg·g^{-1})(L·mg^{-1})$^{1/n}$) and n are Freundlich isotherm coefficients, which are separately related to the adsorption capability and the adsorption intensity.

Eq. S7. Equation for the D–R model.

$$\ln q_e = \ln q_m - k_D \varepsilon^2$$

In this model, q_m (mg·g^{-1}) and k_D (mol^2·kJ^{-2}) are the D–R isotherm constants related to the maximum adsorption quantity and the mean adsorption free energy, respectively.

Eq. S8. Equation for the Polanyi potential ε.

$$\varepsilon = RT \ln\left(1 + \frac{1}{C_e}\right)$$

In this model, R (8.314 J·mol^{-1}·K^{-1}) is the molar gas constant, and T is the absolute temperature expressed in K.

Eq. S9. Equation for the mean adsorption free energy.

$$E = \frac{1}{\sqrt{2k_D}}$$

Eq. S10. Equation for the Temkin model.

$$q_e = \frac{RT}{b}\ln k_T + \frac{RT}{b}\ln C_e$$

In this model, b (equal to -ΔH, kJ·mol^{-1}) denotes the adsorption heat, and k_T (L·mg^{-1}) is the Temkin isotherm constant.

Eq. S11. Equation for the Gibb's free energy ΔG^0.

$$\Delta G^0 = -RT \ln Kq$$

In this equation, R (8.314 J·mol^{-1}·K$^{-1)}$ is the molar gas constant, T is the absolute temperature expressed in K, and K_q (L·g^{-1}) is the distribution coefficient of an

adsorbent that equals to $q_e \cdot C_e^{-1}$.

Eq. S12. Equation for the ln*Kq*.

$$\ln Kq = \frac{\Delta S^0}{R} - \frac{\Delta H^0}{RT}$$

Eq. S13. Equation for the pseudo-first-order kinetic model.

$$\log(q_e - q_t) = \log q_e - \frac{k_1}{2.303} t$$

In this model, k_1 (min^{-1}) represents the kinetic rate constant of the pseudo-first-order adsorption.

Eq. S14. Equation for the pseudo-second-order kinetic model.

$$\frac{t}{q_t} = \frac{1}{k_2 q_e^2} + \frac{1}{q_e} t$$

In this model, k_2 ($g \cdot mg^{-1} \cdot min^{-1}$) denotes the rate constant of the pseudo-second-order adsorption.

Eq. S15. Equation for the Elovich kinetic model.

$$q_t = \frac{1}{\beta} \ln(\alpha\beta) + \frac{1}{\beta} \ln t$$

In this model, α ($mg \cdot g^{-1} \cdot min^{-1}$) refers to the initial adsorption rate, and β ($g \cdot mg^{-1}$) represents the Elovich desorption constant.

Eq. S16. Equation for the intra-particle diffusion model.

$$q_t = k_{id} t^{0.5} + C_i$$

In this model, k_{id} ($mg \cdot g^{-1} \cdot min^{-1/2}$) denotes the kinetic rate constant of the intra-particle diffusion at stage i, and C_i is a constant whose value directly affects the boundary layer thickness of molecular diffusion.

Table S1. Chemical composition of FDG gypsum confirmed by XRF analysis.

Material	Content/ wt.%										
	Ca	S	Si	Mg	Al	F	Fe	K	Na	Cl	P
FGD gypsum	59.78	26.17	4.18	4.03	1.81	1.34	1.29	0.57	0.35	0.34	0.14

Table S2. Chemical composition of FDG gypsum confirmed by EDX analysis.

Material	Content/ wt.%						
	O	Ca	S	Mg	Si	Al	Fe
FGD gypsum	55.77	24.43	13.24	3.12	1.68	0.91	0.85

Table S3. Chemical composition of the purified FDG gypsum confirmed by EDX analysis.

Material	Content/ wt.%		
	O	Ca	S
The purified FGD gypsum	58.15	22.27	19.58

Table S4. Isotherm parameters of four different models for the adsorption of CR and RhB onto $MoO_2/CaSO_4$ composites.

Isotherm models/parameters	CR	RhB
Langmuir		
q_{max} (mg·g^{-1})	853.54	86.38
k_L (L·mg^{-1})	0.0151	0.1913
R^2	0.9979	0.9912
R_L	0.027-0.143	0.022-0.054
Freundilich		
k_F ((mg·g^{-1})(L·mg^{-1})$^{1/n}$)	100.33	49.55
n	3.0252	9.1466
R^2	0.8587	0.8498
D-L		
q_m (mg·g^{-1})	703.42	70.25
k_D (mol^2·kJ^{-2})	1.22 x 10^{-4}	2.13 x 10^{-7}
E (kJ·mol^{-1})	0.0433	1.2654
R^2	0.8944	0.7564
Temkin		
k_T (L·mg^{-1})	0.21	646.77
b (kJ·mol^{-1})	0.0144	0.3423
R^2	0.9653	0.8501

Table S5. An adsorptive capacity comparison of the $MoO_2/CaSO_4$ composite with other adsorbents.

Adsorbents	q_{max} (mg·g^{-1})	References
Activated carbon	6.7 (CR)<br>39.22 (RhB)	[40, 41]
Jute stick powder	35.7 (CR)<br>87.7 (RhB)	[42]
Kaolinite	22.99 (CR)<br>46.08 (RhB)	[43, 44]
α-MoO_3/polyaniline composite	76.22 (CR)<br>36.36 (RhB)	[23]
$MoO_2/CaSO_4$ composite	853.54 (CR)<br>86.38 (RhB)	This study

Table S6. Thermodynamic parameters for the adsorption of CR and RhB onto $MoO_2/CaSO_4$ composites.

Samples	ΔH^0, kJ·mol^{-1}	ΔS^0, J·mol^{-1}·K^{-1}	ΔG^0, kJ·mol^{-1}					R^2
			20 °C	25 °C	30 °C	35 °C	40 °C	
CR	-22.31	-13.02	-18.11	-18.44	-18.33	-18.02	-17.22	0.9221
RhB	40.11	193.25	-16.19	-17.63	-18.64	-19.01	-20.22	0.9755

Table S7. Kinetic parameters of four different models for the adsorption of CR and RhB onto $MoO_2/CaSO_4$ composites.

Kinetic models/Parameters	CR	RhB
Pseudo-first-order		
q_e (exp) ($mg \cdot g^{-1}$)	750.63	66.64
q_e (cal) ($mg \cdot g^{-1}$)	522.60	23.22
k_1 (min^{-1})$\cdot 10^{-3}$	5.20	6.03
R^2	0.8875	0.9064
Pseudo-second-order		
q_e (cal) ($mg \cdot g^{-1}$)	746.27	66.85
k_2 ($g \cdot mg^{-1} \cdot min^{-1}$)$\cdot 10^{-4}$	0.25	9.40
R^2	0.9508	0.9992
Elovich		
α ($mg \cdot g^{-1} \cdot min^{-1}$)	239.83	46.75
β ($g \cdot mg^{-1}$)$\cdot 10^{-3}$	11.08	116.95
R^2	0.8007	0.9225
Intra-particle diffusion		
k_{1d} ($mg \cdot g^{-1} \cdot min^{-1/2}$)	126.93	12.31
C_1	0	0
$(R_1)^2$	1.0000	1.0000
K_{2d} ($mg \cdot g^{-1} \cdot min^{-1/2}$)	18.64	3.77
C_2	266.77	20.20
$(R_2)^2$	0.8897	0.9124
K_{3d} ($mg \cdot g^{-1} \cdot min^{-1/2}$)	6.61	0.53
C_3	568.79	53.79
$(R_3)^2$	0.9492	0.9784
